# Recurrent de novo *WFS1* pathogenic variants in Chinese sporadic patients with nonsyndromic sensorineural hearing loss

**DOI:** 10.1002/mgg3.1367

**Published:** 2020-06-22

**Authors:** Jing Guan, Hongyang Wang, Lan Lan, Yusen Wu, Guohui Chen, Cui Zhao, Dayong Wang, Qiuju Wang

**Affiliations:** ^1^ College of Otolaryngology Head and Neck Surgery Chinese PLA Institute of Otolaryngology Chinese PLA General Hospital Chinese PLA Medical School Beijing China; ^2^ National Clinical Research Center for Otolaryngologic Diseases Chinese PLA General Hospital Chinese PLA Medical School Beijing China; ^3^ State Key Lab of Hearing Science Ministry of Education Chinese PLA General Hospital Chinese PLA Medical School Beijing China; ^4^ Beijing Key Laboratory of Hearing Impairment Prevention and Treatment Chinese PLA General Hospital Chinese PLA Medical School Beijing China

**Keywords:** de novo mutation, nonsyndromic hearing loss, *WFS1*

## Abstract

**Background:**

Hereditary hearing loss (HL) is heterogeneous in terms of their phenotypic features, modes of inheritance, and causative gene mutations. The contribution of genetic variants to sporadic HL remains largely expanding. Either recessive or de novo dominant variants could result in an apparently sporadic occurrence of HL. In an attempt to find such variants we recruited 128 Chinese patients with sporadic nonsyndromic sensorineural HL (NSHL) and performed targeted deafness multigene sequencing in these unrelated trios‐families to elucidate the molecular basis.

**Methods:**

We analyzed a total of 384 available members (probands and their two parents) from 128 unrelated Chinese families presenting with bilateral sensorineural HL, in which previous screening had found no mutations with the *GJB2*, *SLC26A4*, and *MT‐RNR1* genes. We used a targeted genomic enrichment platform to simultaneously capture exons, splicing sites, and immediate flanking intron sequences of 127 known deafness genes. Sanger sequencing was used to identify probands and their two parents segregating causative variants in the candidate gene.

**Results:**

We observed that two heterozygous de novo *WFS1* mutations in exon 8: c.2051C>T (p.A684V) and c.2590G>A (p.E864K) in five families. The two de novo *WFS1* mutations were found in 3.9% (5/128) of sporadic HL patients. We found that four of the five patients had the same de novo p.A684V mutation, and their audiograms showed symmetrical bilateral and profound sensorineural hearing impairments at all frequencies, but only the proband with de novo p.E864K mutation demonstrated significantly bilateral moderate low–mid frequency sensorineural HL. Our data suggest that this *WFS1* p.A684V is likely to be a de novo mutational hot spot.

**Conclusions:**

We found 3.9% (5/128) of sporadic NSHL is caused by de novo* WFS1* mutations. Our data provide that the de novo p.E864K mutation is first identified and de novo p.A684V mutation is likely to be a mutational hot spot in *WFS1*. It is the first study to highlight that *WFS1* gene with the two de novo mutations has been indicated to classify the distinct hearing impairment phenotypes. Furthermore, de novo p.A684V serves as a *WFS1* mutational hot spot that was found in the Chinese population with sporadic childhood NSHL, and our study also provides pointers toward the necessity for sequencing of asymptomatic parents of a sporadic case with an apparent dominant pathogenic variant.

## Graphical abstract

We found 3.9% of sporadic nonsyndromic sensorineural hearing loss is caused by de novo WFS1 mutations.Our data provide that the de novo p.E864K mutation is first identified and de novo p.A684V mutation is likely to be a mutational hot spot in WFS1.

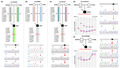

## 1 INTRODUCTION

Hereditary hearing loss (HHL) is a prevalent sensorineural disorder and also a key abnormality in many syndromes. It is estimated that 70% of the genetic forms of HL are nonsyndromic and the remaining 30% are syndromic (Shearer, Hildebrand, & Smith, 1993). HHL is heterogeneous in terms of inheritance modes and causative gene mutations. Variations in many genes implicated in causes of both autosomal dominant (AD) and autosomal recessive (AR) inheritance patterns HL. Genetic molecular diagnostics are increasing our understanding of the molecular physiology of hearing and its loss. Advances in HL diagnosis enabled by genomic technologies are changing the evaluation of patients with HL. In this study, we recruited 128 Chinese patients with sporadic nonsyndromic sensorineural hearing loss (NSHL) and performed targeted deafness multigene sequencing (including 81 known nonsyndromic HL causing genes and 66 known syndromic HL causing genes listed in Table S1) in these unrelated trios‐families to elucidate the molecular basis. We found de novo *WFS1* (MIM *606201, NM_006005.3) mutations in 3.9% (5/128) of NSHL. We identified a heterozygous identical de novo *WFS1* mutation (c.2051C>T/p. A684V) occurring independently in four families and another heterozygous de novo mutation (c.2590G>A/7p. E864K) in a proband. For the first time, our data suggest that *WFS1* de novo mutations are a common cause and A684V might occur as de novo change at a mutational hot spot in the Chinese population with sporadic childhood NSHL.

## 2 METHODS

### 2.1 Ethical compliance

This study was approved by the Committee of Medical Ethics of Chinese People's Liberation Army (PLA) General Hospital. We obtained written informed consent from all the participants in this study. Written informed consent was obtained from the next of kin on the behalf of the minors/children who participants involved in this study.

### 2.2 Clinical data

Initially, we analyzed a total of 384 available members (probands and their two parents) from 128 unrelated Chinese families presenting with bilateral sensorineural HL, in which previous screening had found no mutations with the *GJB2* (MIM *121011), *SLC26A4* (MIM *605646), and *MT‐RNR1* (MIM *561000) genes. We identified *WFS1* mutations in five of these probands and the five families are described here in detail. Five two‐generation Chinese families (CN‐1707877, CN‐1507315, CN‐1707650, CN‐1808062, and CN‐1707661) with NSHL were recruited from the Department of Otolaryngology and Head and Neck Surgery at the Chinese PLA General Hospital. The subjects underwent a full medical history and comprehensive audiological evaluation, including otoscopic examination, pure‐tone audiometry, tympanometry, acoustic reflex; Temporal bone Computed Tomography (CT) scans were also performed. The proband in family CN‐1707661 additionally underwent distortion product evoked otoacoustic emissions (DPOAEs), auditory brainstem responses (ABRs), electrocochleogram, colic vestibular evoked myogenic potential (cVEMP) testing, and other systematic examination during hospitalized medication treatment her hearing impairment.

Air conduction (AC) thresholds were bilaterally determined at octave frequencies of 0.125–8.0 kHz. The HL range was described based on the parameters: low frequency, <500 Hz; mid frequency, 501–2000 Hz; and high frequency >2000 Hz (Shearer et al., 1993). Hearing levels were labeled subtle (16–25 dB), mild (26–40 dB), moderate (41–70 dB), severe (71–95 dB), or profound (95 dB) (Kim et al., 2015).

### 2.3 Targeted gene capture and high throughput sequencing

Genomic DNAs of the probands, their parents were extracted from peripheral blood samples using the Blood DNA kit (TIANGEN BIOTECH). We used a targeted genomic enrichment platform to simultaneously capture exons, splicing sites, and immediate flanking intron sequences of 127 known deafness genes point mutations, micro‐indels and duplications (<20 bp) could be detected simultaneously. Targeted gene capture and high throughput sequencing have been described in detail previously. Sanger sequencing was used to identify probands and their two parents segregating causative variants in the candidate gene (Guan et al., 2018). The data were not analyzed for copy number variants (CNVs).

### 2.4 Haplotype analysis

DNA analysis of up to 750,000 single nucleotide polymorphisms (SNPs)‐based parental testing was carried out on three families (CN‐1707650, 1808062, and 1507315), confirming that they are the patients biological parents. We performed haplotype analysis in the three families, harboring the p.A684V mutation, using 12 SNP markers flanking *WFS1* (Table S2).

## 3 RESULTS

The pedigrees of the five families here all present no family history with same symptom. All probands were from independent families with nonconsanguineous and healthy Chinese parents. Each proband of these families was pretested for the *GJB2*, *SLC26A4*, and *MT‐RNR1* genes and was negative for these genes.

**FIGURE 1:**
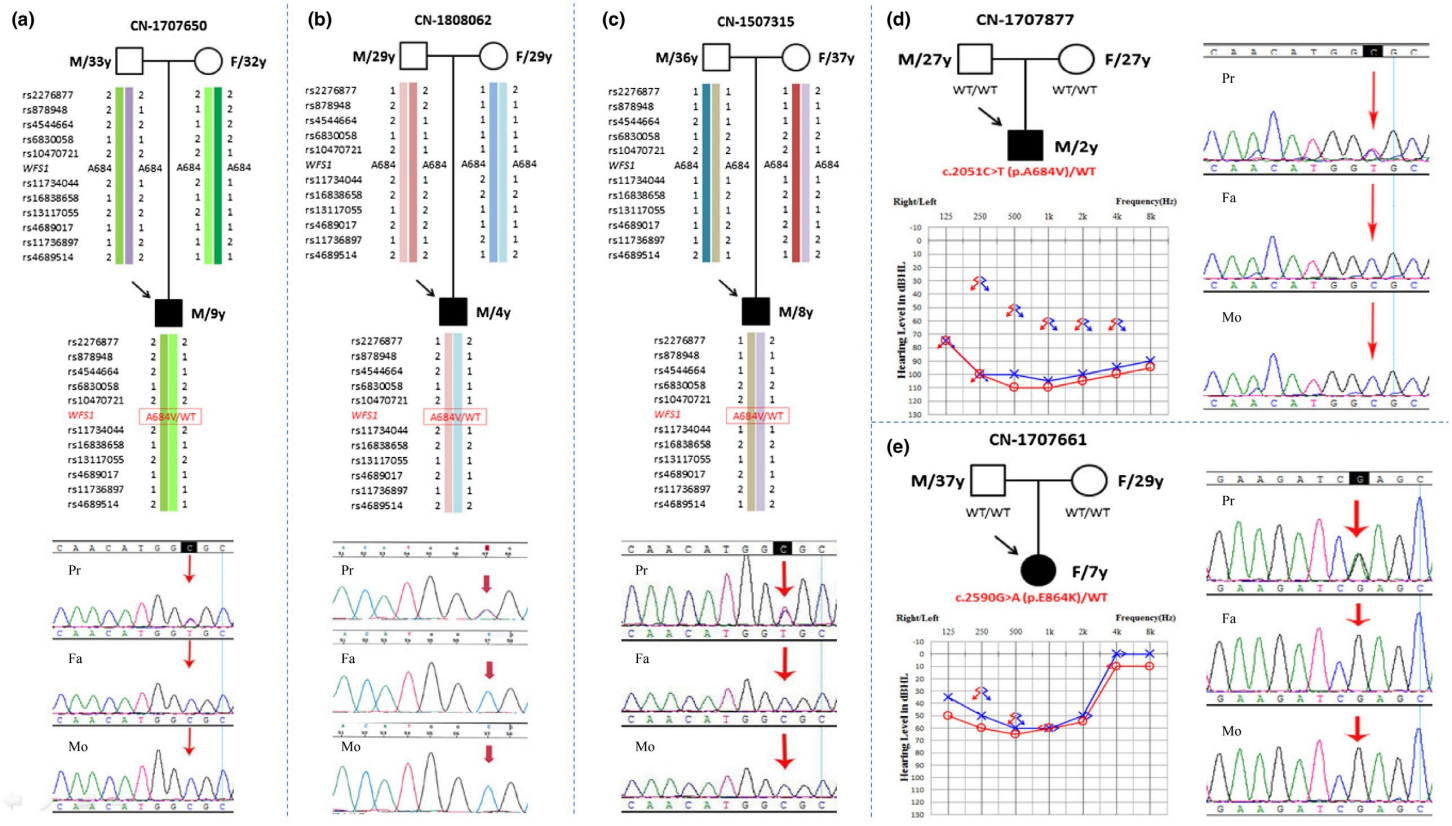
Pedigree, haplotype analysis, audiogram, and mutational analysis of families with de novo *WFS1* mutations. (a–c): Haplotype analysis in three families with the recurrent de novo heterozygous *WFS1* p.A684V and Sanger sequence chromatograms. (d–e): The pedigree and audiograms of the affected subjects with de novo heterozygous *WFS1* p.A684V (NM_006005.3: c. 2051C>T) and de novo heterozygous *WFS1* p.E864K (NM_006005.3: c. 2590G>A) and DNA sequencing profile. The results of pure‐tone audiometry exhibited bilateral profound hearing loss in all frequencies for the patient with *WFS1* p.A684V and bilateral moderate hearing loss in low–middle frequencies for the patient with *WFS1* p.E864K. The horizontal axis shows tone frequency (Hz); the vertical axis gives hearing level (dB). Symbols “o” and “x” denote air conduction pure‐tone thresholds at different frequencies in the right and left ear. The age at the time of audiological examination was recorded. (a–e): Sanger sequencing demonstrates the heterozygous mutation in the chromatogram from affected individuals (upper row) and no variant from unaffected parents (lower rows). Symbols “Pr,” “Fa,” and “Mo” denote proband, father, and mother, respectively

**FIGURE 2:**
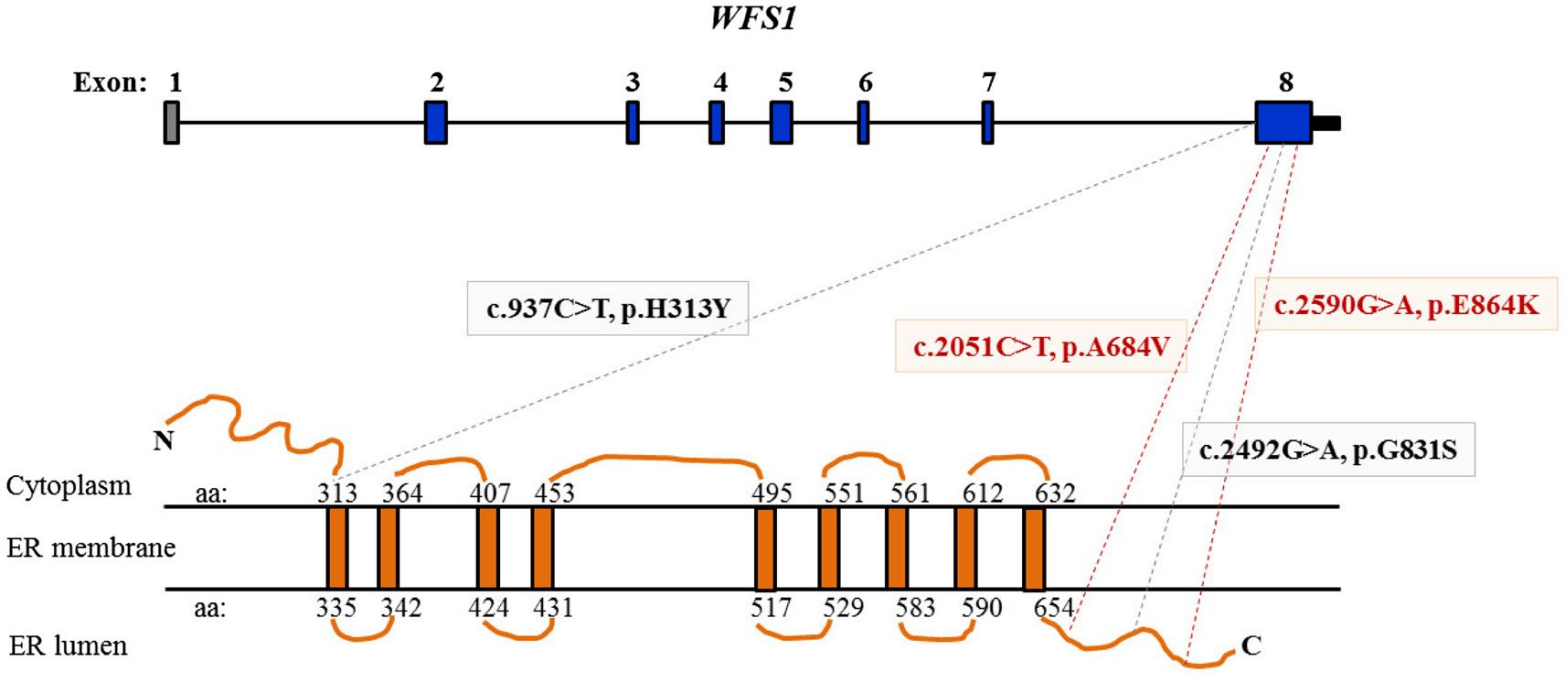
Summary and depiction of *WFS1* de novo mutations. *WFS1* comprises eight exons [NCBI Reference Sequence NM_006005.3]. The first exon is not coding and the largest exon is exon 8. The recurring de novo missense variants of previously reported and our present study located in the exon 8. The mutations reported in our probands are shown in red. Schematic diagram of the WFS1 nine transmembrane domains map. “ER” denotes endoplasmic reticulum

We identified two previously reported heterozygous *WFS1* mutation (NM_006005.3: c.2051C>T, p.A684V and c.2590G>A, p.E864K) in exon 8 (Figure 2) and no candidate pathogenic variants in the other 127 deafness genes in the sporadic cases. We then used Sanger sequencing to screen the proband's parents. The results showed that none of their asymptomatic parents had the same genotype as the probands, indicating that p.A684V and p.E864K were de novo occurrence. In addition, we carried out parental testing on the three families with the *WFS1* p.A684V and confirmed that they are the patients’ biological parents, which are strongly support for the de novo variations (Figure 1a–c). Most interestingly, four of the five patients had the identical de novo mutation (c.2051C>T, p.A684V), and their audiograms showed symmetrical bilateral and profound sensorineural hearing impairments at all frequencies and the age of onset varied from 8 months to 2 years (Figure 1d). Furthermore, all the four probands underwent cochlear implantation treatment before age 3 and their language ability improved after surgery.

**FIGURE 3:**
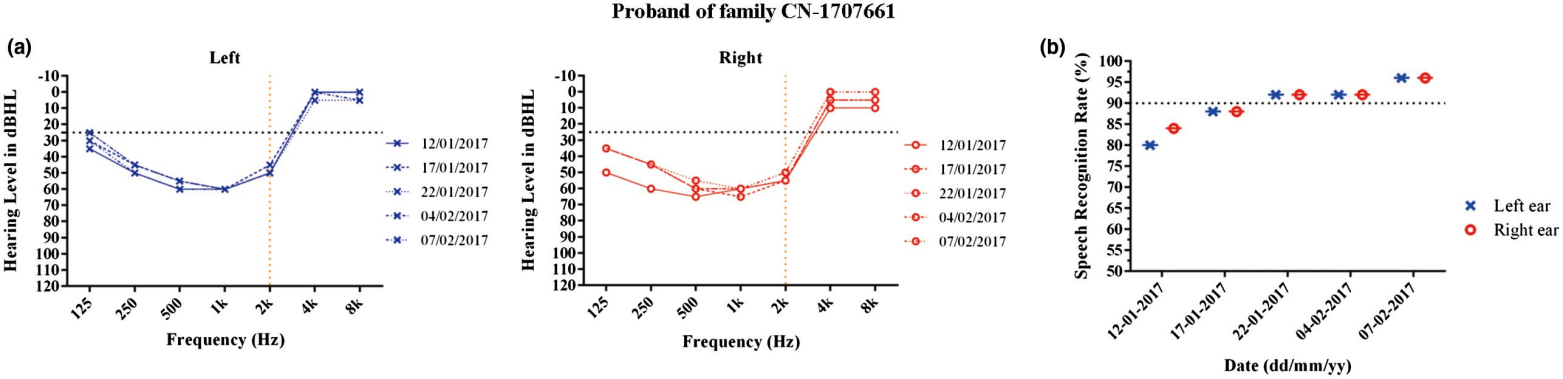
Audiograms information of the 7‐year‐old proband of family CN‐1707661 with *WFS1* de novo c.2590G>A, p.E864K. (a) Audiogram changes of the affected individual during treatment. They exhibited bilateral moderate hearing loss in low–middle frequencies. The horizontal axis shows tone frequency (Hz); the vertical axis gives hearing level (dB). Symbols “o” and “x” denote air conduction pure‐tone thresholds at different frequencies in the right and left ear. The horizontal black dotted line shows that hearing is considered abnormal if beyond 25 dB thresholds. The vertical orange dotted line shows the boundary between middle and high frequencies. The individual had no significant hearing threshold changes after treatment. (b) The individual had better hearing speech recognition rate after treatment, thus allowing communicating and connecting more easily. The horizontal black dotted line shows that hearing speech recognition rate >90% correlates better with hearing sensitivity

However, only the proband in family CN‐1707661 was a de novo heterozygous for p.E864K and demonstrated significantly bilateral moderate low–mid frequency sensorineural HL audiometric configuration, which does not impact speech and language (Figure 1e). So, the proband was a 7‐year‐old girl whose parents just noticed hearing impairment. She exhibited normal latency and amplitude of ABR and cVEMP waves (Figure S1). Temporal bone CT scans were normal. The patient was treated with a combination therapy of local steroids, Ginkgo biloba extract (EGb), Methyl cobalamin and Monosialoganglioside, which especially improve her hearing speech recognition rate (>90%) (Figure 3).

We found that these patients with de novo mutation (p.A684V) had significantly worse HL than the patient with de novo mutation (p.E864K) (Table 1). The telephone follow‐up calls were immediately conducted after we identified the disease‐causing *WFS1* gene. All the parents reported that their children had no visual problems but details of visual acuity were not recorded. They also reported no vestibular disorders, no progression in HL and no diabetes. Table 1 summarizes the clinical and genetic findings for the present study five individuals with de novo *WFS1* mutations and previously reported cases with other de novo mutations or the two same mutations. But unlike two previously reported *WFS1* mutation (p.A684V and p.E864K) cases, none of our patients had Wolfram‐like syndrome and sensorineural HL is the only disease phenotype.

**TABLE 1:** Clinical features in five probands with de novo *WFS1* mutation in this study and the published reports of the same mutation and other de novo mutations

Patient/Family No.	Origin	Family history	Tested age/Sex	*WFS1* mutation			Auditory phenotype			Other phenotypes		Reference
				HGVS. c	HGVS. p	Inheritance	Onset	Severity	Configuration	WS	WLS	
CN‐1707877	China	Sporadic	2 years/M	c.2051C>T	p. A684V	De novo	2 years	Profound	Flat	(−)	(−)	Present study
CN‐1507315	China	Sporadic	8 years/M	c.2051C>T	p. A684V	De novo	1 year	Profound	Flat	(−)	(−)	Present study
CN‐1707650	China	Sporadic	9 years/M	c.2051C>T	p. A684V	De novo	2 years	Profound	Flat	(−)	(−)	Present study
CN‐1808062	China	Sporadic	4 years/M	c.2051C>T	p. A684V	De novo	8 months	Profound	Flat	(−)	(−)	Present study
WS28	French	Sporadic	4 years/F	c.2051C>T	p. A684V	De novo	20 months	Severe	NA	(−)	(+)	Chaussenot et al. (2015)
Family11/1	French	Sporadic	6 years/F	c.2051C>T	p. A684V	De novo	1.5 years	NA	NA	(−)	(+)	Grenier et al. (2016)
Family13/1	French	Sporadic	50 years/M	c.2051C>T	p. A684V	De novo?	3 years	NA	NA	(−)	(+)	Grenier et al. (2016)
Family #7‐1	Japan	Sporadic	15 years/F	c.2051C>T	p. A684V	De novo?	0	Profound	Flat	(−)	(−)	Kobayashi et al. (2018)
Family 81	US/Caucasian	Sporadic?	55 years/M	c.2051C>T	p. A684V	De novo?	3 years	Profound	NA	(−)	(+)	Rendtorff et al. (2011)
Family KW200128	Sweden	AD	NA/F	c.2051C>T	p. A684V	Maternally inherited	Childhood	Severe	NA	(−)	(+)	Rendtorff et al. (2011)
Family NSDF916	UK	AD	69 years/F	c.2051C>T	p. A684V	Maternally inherited	Congenital	Severe to profound	NA	(−)	(+)	Rendtorff et al. (2011)
Family NSDF2032	US/Caucasian	AD	54 years/F	c.2051C>T	p. A684V	Maternally inherited	Congenital	NA	NA	(−)	(+)	Rendtorff et al. (2011)
Family NSDF1865	US/Caucasian	AD	46 years/F	c.2051C>T	p. A684V	Paternally inherited	1.5 years	Severe to profound	NA	(−)	(+)	Rendtorff et al. (2011)
Family 3	Italian	NA	22/F	c.2051C>T	p. A684V	NA	14 years	NA	NA	(+)	(−)	Tessa et al. (2001)
CN‐1707661	China	Sporadic	7 years/F	c.2590G>A	p. E864K	De novo	7 years	Moderate	Low/Mid‐frequency	(−)	(−)	Present study
Family‐V:3	Danish	AD	19 years/F	c.2590G>A	p. E864K	Paternally inherited	4 years	Moderate	Low‐frequency	(−)	(+)	Eiberg et al. (2006)
Family #18‐1	Japan	AD	29 years/F	c.2590G>A	p. E864K	Maternally inherited	3 years	Profound	Flat	(−)	(+)	Kobayashi et al. (2018)
Family #19‐1	Japan	AD	6 years/F	c.2590G>A	p. E864K	Paternally inherited	3 years	Moderate	Low‐frequency	(−)	(−)	Kobayashi et al. (2018)
Family #2	Japan	AD	NA	c.2590G>A	p. E864K	Maternally inherited	NA	Moderate to severe	Low‐frequency	(−)	(−)	Fukuoka et al. (2007)
Family #3	Japan	AD	NA	c.2590G>A	p. E864K	Maternally inherited	NA	Moderate to severe	Low‐frequency	(−)	(−)	Fukuoka et al. (2007)
Family‐III‐2	French	AD	27 years/M	c.2590G>A	p. E864K	Maternally inherited	Childhood	Moderate	U‐shape	(−)	(+)	Valero et al. (2008)
Family D	Finland	Sporadic	NA/F	c.2492G>A	p. G831S	De novo	1.6 years	Profound	NA	(−)	(−)	Sanna et al. (2014)
EE33 II:2	Danish	Sporadic	10 years/F	c.937C>T	p.H313Y	De novo	2 months	Profound	NA	(−)	(+)	Hansen et al. (2005)
EE77 II:1	Danish	Sporadic	24 years/M	c.937C>T	p.H313Y	De novo	Congenital	Profound	NA	(−)	(+)	Hansen et al. (2005)

Note

‘?’, DNA samples could not be obtained from their parents; “+”, present; “−”, not present; NA, not available; Fill yellow color for the mutation NM_006005.3: c.2051C>T (p.A684V), green color for the mutation NM_006005.3: c.2590G>A (p.E864K), and light gray for other *WFS1* de novo mutations.

Abbreviation: AD, autosomal dominant.

## 4 DISCUSSION

The function of *WFS1* is very important to the auditory system. *WFS1* contains eight exons and encodes a transmembrane protein (wolframin) that is predominantly localized in the endoplasmic reticulum (ER) (Figure 2). It plays an important role in maintaining correct calcium levels in cell and its lack of function induces apoptotic input signaling in the ER. Individuals with mutations in *WFS1* present with three different phenotypes: AR Wolfram syndrome (WS, MIM #222300) featuring sensorineural HL, diabetes mellitus (DM), optic atrophy (OA) and various forms of neurologic impairment, diabetes insipidus and urinary dysfunctions, AD progressive HL with OA or impaired glucose regulation also called Wolfram‐like syndrome (WLS, MIM #614296), and DFNA6/14/38‐associated AD nonsyndromic sensorineural HL (NSHL, MIM #600965) (Grenier et al., 2016; Niu et al., 2017). No plausible functional explanation has been found to explain the vast differences in clinical presentations and patterns of inheritance. Notably, HL is a key abnormality in the three *WFS1*‐associated disorders. To date, there are more than 320 variants in *WFS1* identified in multiple independent families from different ethnic backgrounds (https://www.ncbi.nlm.nih.gov/dbvar). Most *WFS1* mutations are unique to an individual or a family. However, only a few of the included mutations may be “hot spot” (Niu et al., 2017) and true de novo mutations are even uncommon.

In the present study, multigene testing panels sequencing strategy was used to find the disease‐causing gene of 128 unrelated Chinese families with NSHL, and we identified that *WFS1* mutations account for about 3.9% (5/128) of NSHL in Chinese patients negative for the *GJB2*, *SLC26A4*, and *MT‐RNR1* mutations. We observed that two heterozygous de novo *WFS1* mutations in exon 8: c.2051C>T (p.A684V) and c.2590G>A (p.E864K) in five families. Consistent with previous reports, the *WFS1* mutations are mainly missense mutations in exon 8 and present in the C‐terminal part of the protein for NSHL (Eiberg et al., 2006; Tessa et al., 2001).

*WFS1* mutations are responsible for various phenotypes of HL including at low, high or all frequencies (Bai et al., 2014; Kytovuori, Hannula, Maki‐Torkko, Sorri, & Majamaa, 2017). Interestingly, our results indicate that four of the five patients had the same de novo p.A684V mutation, and their audiograms showed symmetrical bilateral and profound sensorineural hearing impairments at all frequencies, but only the proband with de novo p.E864K mutation demonstrated significantly bilateral moderate low–mid frequency sensorineural HL. Table 1 summarizes the clinical features of the 14 *WFS1* c.2051C>T (p.A684V) mutations reported to date, which are associated with similarly severe to profound flat HL and age of onset in four ethnic groups worldwide(Chaussenot et al., 2015; Grenier et al., 2016; Kobayashi et al., 2018; Rendtorff et al., 2011; Tessa et al., 2001). So, all the four probands with p.A684V mutation underwent cochlear implantation treatment before age 3 and their language ability improved after surgery. Of note, the *WFS1* p.A684V de novo mutation was also found in two French WLS sporadic patients (Chaussenot et al., 2015; Grenier et al., 2016). Moreover, the same de novo mutation has also been hypothesized in a Japanese NSHL (Kobayashi et al., 2018), a French WLS (Grenier et al., 2016), and an US/Caucasian sporadic cases (Rendtorff et al., 2011). These data suggest that this *WFS1* p.A684V is likely to be a de novo mutational hot spot (9/14). Furthermore, we also observed another *WFS1* de novo mutation p. E864K resulted in nonsyndromic low‐frequency HL. Due to maintenance of language‐frequency hearing, the affected subject retains excellent understanding of speech. So, the patient's parents were not aware of her hearing impairment until she was 7 years old. Subsequently, the parents actively sought treatment for their child and a combination therapy of local steroids, Ginkgo biloba extract (EGb), Methyl cobalamin and Monosialoganglioside, and had therapeutic effect on her speech recognition rate (Figure 3), thus allowing communicating and connecting more easily. To the best of our knowledge, this is the first to show de novo p. E864K mutation. The mutation summary data (Table 1) suggest that the majority patients (5/7) were moderate to severe low‐frequency HL. Other two independent research groups from Japan reported that all three clinically diagnosed nonsyndromic low‐frequency HL patients had *WFS1* p. E864K mutation (Fukuoka, Kanda, Ohta, & Usami, 2007; Kobayashi et al., 2018). However, the site mutation may frequently occur inherited (6/7) rather than de novo onset. Although no plausible explanation has been found to classify the distinct hearing impairment phenotypes by genotypes, our data above draw some noteworthy features that it seems to have clear‐cut genotype–phenotype correlation between p.A684V and p.E864K mutations.

Besides, two additional heterozygous mutations have also previously been reported in a de novo state. One *WFS1* pathogenic variant p.G831S (c.2492G>A) was found in a Finland sporadic case with NSHL and another p.H313Y (c.937C>T) mutation was shared by two unrelated Danish sporadic patients with WLS (Hakli, Kytovuori, Luotonen, Sorri, & Majamaa, 2014; Hansen et al., 2005). To our knowledge, together with our present study, there are only four de novo mutations in *WFS1* reported in different racial backgrounds from Asian, European, and US/Caucasian ancestries (Table 1). These data suggest that, de novo *WFS1* mutation may be a relatively common and an important cause of NSHL. Interestingly, de novo p.A684V mutation has been previously identified in different ethnic groups, suggesting that this site is recurrent and possibly appears to be a mutational hot spot in *WFS1*.

*WFS1* heterozygous mutations are also a common cause of WLS, which is characterized by AD inherited sensorineural HL with variable OA, DM, and/or psychiatric illness. The two heterozygous mutation p. A684V and p. E864K in *WFS1* gene have been documented to cause WLS disease. The age of onset of OA varied rang 11–43 years (Grenier et al., 2016). However, no validated data are available on the age of onset of DM or neurologic abnormalities. At present, the oldest patient reported with NSHL‐associated *WFS1* heterozygous mutations p. A684V or p.E864K is 15 years old (Table 1), and this patient showed no abnormalities in other phenotypes of WLS (Kobayashi et al., 2018). In our study, the age of the NSHL affected individuals was younger and they described no signs of visual or neural by our telephone following up. However, our patients with the pathogenic variant are at risk of developing other phenotypes of late‐onset WLS in older ages. So longer follow‐up studies evaluating changes in the risk are important.

In summary, we identified two previously reported *WFS1* mutations (p. A684V and p. E864K) in five unrelated Chinese families. Importantly, we found 3.9% (5/128) of sporadic NSHL is caused by de novo *WFS1* mutations. Our data provide that the de novo p.E864K mutation is first identified and de novo p.A684V mutation is likely to be a mutational hot spot in *WFS1*. Furthermore, we show that, even if a de novo mutation for hereditary HL is uncommon event, Sanger sequencing of asymptomatic parents must be performed in a sporadic case with an apparent dominant pathogenic variant.

## CONFLICT OF INTEREST

The authors declare that they have no conflict of interest.

## AUTHOR CONTRIBUTION

QW and JG conceived and designed the experiments and wrote the paper. JG and HW performed the experiments. JG analyzed the data. HW, LL, YW, GC, CZ, and DW contributed reagents/materials/analysis tools. JG, HW, and QW critically read and discussed the manuscript. All authors read and approved the final manuscript. We obtained written informed consent for publishing all data of this study from all the participants, such as medical data, images, and genetic results. Written informed consent was obtained from the next of kin on the behalf of the minors/children who participants involved in this study.

## Supporting information

Fig S1

Table S1‐S2

## Data Availability

All data are present in the manuscript or additional files.
