# Supplementary figures and images for: Recurrent de novo WFS1 pathogenic variants in Chinese sporadic patients with nonsyndromic sensorineural hearing loss
Source: Mol Genet Genomic Med. 2020 Jun 22;8(8):e1367. doi: 10.1002/mgg3.1367 (PMC7434732; doi:10.1002/mgg3.1367)

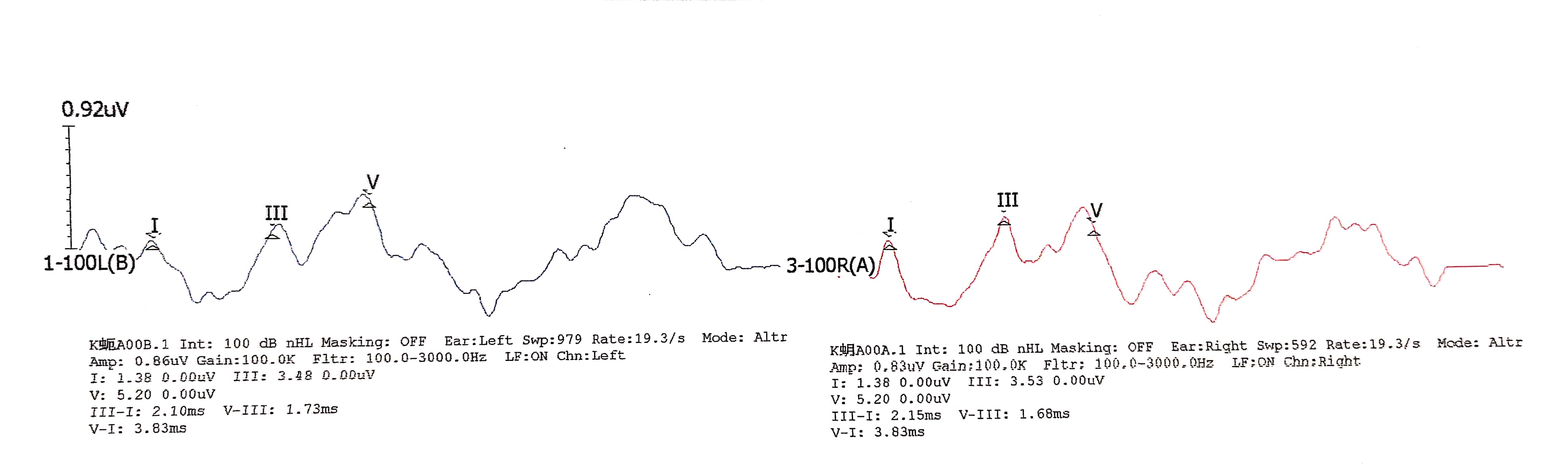

Supplement: Supplementary file 1 — Fig S1 [file MGG3-8-e1367-s001.jpg]
