# Supplementary material for: Recurrent de novo WFS1 pathogenic variants in Chinese sporadic patients with nonsyndromic sensorineural hearing loss
Source: Mol Genet Genomic Med. 2020 Jun 22;8(8):e1367. doi: 10.1002/mgg3.1367 (PMC7434732; doi:10.1002/mgg3.1367)
Supplement: Supplementary file 2 — Table S1‐S2 [file MGG3-8-e1367-s002.docx]

**Table S1. Targeted genomic capturing 127 deafness genes**

| **A** | *ATP2B2, ACTG1, ALMS1* |
| --- | --- |
| **B** | *BSND* |
| **C** | *CDH23,CLRN1,CLDN14,CCDC50,CEACAM16,COCH,CRYM,COL2A1, CIB2*  *COL11A1,COL9A1,COL9A2,COL4A3,COL4A4,COL4A5,CHD7,CACNA1D* |
| **D** | *DFNB31, DFNA5,DIABLO,DIAPH1,DSPP, DIAPH3, DLX5* |
| **E** | *ESRRB,ESPN, EYA1,EYA4, EDNRB,EDN3* |
| **F** | *FOXI1, FGFR3,FGFR1,FGFR2, FGF3* |
| **G** | *GJB2,GJB3,GJB6,GIPC3,GRXCR1,GPSM2,GRHL2,GATA3,GLI3,GPR98* |
| **H** | *HGF, HOXA1, HOXA2* |
| **I** | *ILDR1, IGF1* |
| **K** | *KCNJ10, KCNQ4, KCNQ1,KCNE1* |
| **L** | *LRTOMT, LHFPL5, LOXHD1, LRP2* |
| **M** | *MYO1A,MYO3A,MYO6,MYO7A,MYO15A,MARVELD2,MSRB3,MIR96,MYH14,MYH9*  *MT-RNR1,MT-TS1, MT-TK,MT-TE,MT-TL1, MITF* |
| **N** | *NDP,NLRP3* |
| **O** | *OTOF, OTOG, OTOA, OPA1* |
| **P** | *PCDH15,PTPRQ,POU4F3,POU3F4,PRPS1,PDSS1,PHEX,PAX2,PAX3,PRRX1, PDZD7* |
| **R** | *RDX* |
| **S** | *SLC17A8,SLC26A4,SLC26A5,STRC,SERPINB6,SIX1,SIX5,SMPX,SERAC1,SOX9,SLC19A2*  *SNAI2, SOX10, SOBP, SEMA3E,SMAD4, SLC4A11* |
| **T** | *TMIE,TMC1,TMPRSS3,TECTA,TRIOBP,TPRN,TJP2,TNFRSF11B,TCOF1,TIMM8A* |
| **U** | *USH1C, USH1G,USH2A* |
| **W** | *WFS1* |

**Table S2. Distance and position of SNP marker to *WFS1* A684**

| SNP marker | Genetic_DIS | Physiton_POS |
| --- | --- | --- |
| rs2276877 | 10.6514 | 5851343 |
| rs878948 | 11.2062 | 6024426 |
| rs4544664 | 11.366 | 6074289 |
| rs6830058 | 11.5061 | 6118000 |
| rs10470721 | 11.756 | 6195962 |
| A684 | 12.101 | 6303573 |
| rs11734044 | 12.2108 | 6337829 |
| rs16838658 | 12.3308 | 6375269 |
| rs13117055 | 12.4756 | 6487726 |
| rs4689017 | 12.7503 | 6541542 |
| rs11736897 | 13.2814 | 6645597 |
| rs4689514 | 13.4882 | 6686114 |
